# Home delivery of the communicator for remote monitoring of cardiac implantable devices: A multicenter experience during the covid‐19 lockdown

**DOI:** 10.1111/pace.14251

**Published:** 2021-05-15

**Authors:** Michele Magnocavallo, Alessia Bernardini, Marco Valerio Mariani, Agostino Piro, Massimiliano Marini, Antonino Nicosia, Carmen Adduci, Antonio Rapacciuolo, Davide Saporito, Stefano Grossi, Giuseppe Santarpia, Paola Vaccaro, Roberto Rordorf, Francesco Pentimalli, Giuseppe Giunta, Monica Campari, Sergio Valsecchi, Carlo Lavalle

**Affiliations:** ^1^ Department of Cardiovascular Respiratory Nephrology Anaesthesiology and Geriatric Sciences Sapienza University of Rome Rome Italy; ^2^ Department of Cardiology S. Chiara Hospital Trento Italy; ^3^ Cardiology Unit Giovanni Paolo II Hospital Ragusa Italy; ^4^ Division of Cardiology Department of Clinical and Molecular Medicine, Sapienza University of Rome St. Andrea Hospital Rome Italy; ^5^ Cardiology Unit Università degli Studi di Napoli Federico II Naples Italy; ^6^ Division of Cardiology Infermi Hospital Rimini Italy; ^7^ Cardiology Department Azienda Ospedaliera Ordine Mauriziano Turin Italy; ^8^ Division of Cardiology Department of Medical and Surgical Sciences Magna Graecia University Catanzaro Italy; ^9^ Cardiology Unit Riuniti Hospital P.O. Cervello Palermo Italy; ^10^ Department of Cardiology Fondazione IRCCS Policlinico S. Matteo Pavia Italy; ^11^ Cardiology Unit San Paolo Hospital Savona Italy; ^12^ Boston Scientific Milan Italy

**Keywords:** cardiac implantable electronic device, covid‐19, follow‐up, remote monitoring

## Abstract

**Background:**

During the COVID‐19 pandemic in‐person visits for patients with cardiac implantable electronic devices should be replaced by remote monitoring (RM), in order to prevent viral transmission. A direct home‐delivery service of the RM communicator has been implemented at 49 Italian arrhythmia centers.

**Methods:**

According to individual patient preference or the organizational decision of the center, patients were assigned to the home‐delivery group or the standard in‐clinic delivery group. In the former case, patients received telephone training on the activation process and use of the communicator. In June 2020, the centers were asked to reply to an *ad hoc* questionnaire to describe and evaluate their experience in the previous 3 months.

**Results:**

RM was activated in 1324 patients: 821 (62%) received the communicator at home and the communicator was activated remotely. Activation required one additional call in 49% of cases, and the median time needed to complete the activation process was 15 min [25th‐75th percentile: 10–20]. 753 (92%) patients were able to complete the correct activation of the system. At the time when the questionnaire was completed, 743 (90%) communicators were regularly transmitting data. The service was generally deemed useful (96% of respondents) in facilitating the activation of RM during the COVID‐19 pandemic and possibly beyond.

**Conclusions:**

Home delivery of the communicator proved to be a successful approach to system activation, and received positive feedback from clinicians. The increased use of a RM protocol will reduce risks for both providers and patients, while maintaining high‐quality care.

**Abbreviations**
CIED: Cardiac Implantable Electronic Devices
COVID‐19: Coronavirus Disease 2019
CRT: Cardiac Resynchronization Therapy
ESC: European Society of Cardiology
ICD: Implantable Cardioverter Defibrillator
RM: Remote Monitoring
SARS‐CoV‐2: Severe Acute Respiratory Syndrome‐CoronaVirus‐2.

## 1 INTRODUCTION

In December 2019, an outbreak of pneumonia caused by a novel coronavirus occurred in Wuhan, China.^1^ The virus was identified as severe acute respiratory syndrome‐coronavirus‐2 (SARS‐CoV‐2), which causes Coronavirus disease 2019 (COVID‐19), and triggered a worldwide health emergency.^2^, ^3^, ^4^, ^5^ On March 8th, Italy became the second most severely affected country in the world, and specific restrictions on social contacts were imposed by the Italian government.^6^ In accordance with the European Society of Cardiology (ESC) guidelines for the diagnosis and management of cardiovascular disease during the COVID‐19 pandemic,^7^ patients with ambulatory stable heart failure had to refrain from hospital visits, and cardiology centers were strongly encouraged to conduct follow‐up and provide patients with medical advice by means of telemedicine, in order to prevent viral transmission to patients and healthcare providers.^8^, ^9^, ^10^ These recommendations specifically applied to patients with cardiac implantable electronic devices (CIEDs).^11^, ^12^ For these patients, in‐person office visits could be replaced by remote contact, by using the device information obtained through remote monitoring (RM).^13^, ^14^ Since the beginning of the lockdown phase in Italy, Boston Scientific has communicated to all Italian centers implanting CIEDs its willingness to implement a direct home‐delivery service of the LATITUDE communicator, in order to allow RM of all patients not yet monitored, without requiring access to the hospital. The initiative included delivery of the communicator, the informed consent process, and organization of remote training for patients and the staff of the center, if needed.

Here, we report the results of a questionnaire designed to evaluate the experience of the centers that adhered to the “LATITUDE at home” campaign.

## 2 METHODS

Starting on March 20, the centers adhering to the initiative identified patients with an implanted Boston Scientific CIED with RM capabilities who were not yet enrolled in the LATITUDE platform. The staff of all centers not yet using LATITUDE received remote training in the use of the platform from the Boston Scientific technical support team. Patients were contacted and offered a communicator for RM of their devices. According to individual patient preference or the organizational decision of the center, patients were assigned to the home‐delivery group or the in‐clinic delivery group. In the former case, patients received telephone training on the activation process and use of the communicator; additional phone contacts were made before or after the delivery of the communicator, if deemed necessary or if requested by the patient. In the latter case, an in‐clinic visit was scheduled, during which the communicator was delivered, and training was provided. Each center designed specific pathways for in‐clinic visits, to guarantee patients' and workers' safety. Patients and caregivers were required to wear personal protective equipment, the clinics were sanitized between one visit and the next, the attending nurse or physician underwent COVID‐19 testing periodically or if at‐risk exposure was suspected. All patients were asked to perform a manual transmission upon receipt of the communicator, in order to complete the activation of the system.

In June, the centers were asked to reply to an *ad hoc* questionnaire to evaluate their experience in the period between March 20 and the end of May. The purpose of the questionnaire was to characterize the use of RM at the centers before the lockdown period, to describe the changes introduced during the lockdown and the use of the home‐delivery service, to measure the effectiveness of monitoring in terms of successful activations and number of systems regularly transmitting data, to assess the workload generated, and to collect feedback on the delivery method. The questionnaire consisted of 33 questions (see Supplementary material for details).

### 2.1 Statistical analysis

In the present report, continuous data are expressed as medians and interquartile ranges. Categorical data are expressed as percentages. Differences in proportions were compared by means of Chi‐square analysis or Fisher's exact test, as appropriate. A *p*‐value < .05 was considered significant for all tests. All statistical analyses were performed by means of R: a language and environment for statistical computing (R Foundation for Statistical Computing, Vienna, Austria).

## 3 RESULTS

### 3.1 Participating centers

During the lockdown period, RM by means of the LATITUDE system was activated in 1324 patients at 49 Italian arrhythmia centers; a complete list of participating centers is reported in Appendix. The centers which participated in the survey accounted for 13% of all 372 arrhythmia centers operating in Italy in 2019. The participating centers were located in 13 Italian regions. Thirteen centers were located in regions with a high incidence of COVID‐19 cases (>3.0 confirmed cases per 1000 population: Lombardy, Piedmont, Veneto, Trentino, Emilia, Liguria), 16 in regions with intermediate incidence (from 1.0 to 3.0 confirmed cases per 1000 population: Lazio, Tuscany, Puglia), and 20 in regions with a low incidence (<1.0 confirmed case per 1000 population: Campania, Calabria, Sicily, Sardinia). In 17 centers, 50 or more patients were being remotely monitored via the LATITUDE system before the lockdown period, in 24 centers the number was less than 50, and eight centers were not using the LATITUDE system for RM prior to the lockdown period. At the centers, remote data were routinely reviewed by the physician, or by a nurse and a physician in accordance with a ‘Primary Nursing’ model (Table 1). In addition to scheduled remote interrogations, RM was frequently used to detect device performance issues and arrhythmias or to monitor the patient's clinical status, by enabling dedicated alerts (Table 1).

**TABLE 1:** Survey questions on the remote monitoring (RM) service implemented at the centers, changes during the lockdown period and clinicians' opinions of RM

Remote monitoring at the centers that responded to the survey	
Professionals involved in the management of remote monitoring	‐ Physicians 45%
	‐ Nurses and Physicians 55%
Automatic alerts enabled	‐ Device performance 77%
	‐ Arrhythmias/Clinical status 70%

Changes during the lockdown period	
Frequency of scheduled remote interrogations	‐ Unchanged 58%
	‐ Increased 36%
	‐ Decreased 6%
Programming of alerts	‐ Yes 6%
	‐ No 94%
In‐clinic scheduled visits	‐ All confirmed 29%
	‐ All cancelled 17%
	‐ Only in specific situations or for patients at higher risk 54%
Additional personnel needed to manage remote monitoring	‐ Yes 22%
	‐ No 78%

Clinician feedback on remote monitoring at the end of the lockdown period	
Was remote monitoring effective in managing patients during the lockdown period?	‐ Yes 98%
	‐ No 2%
Did remote monitoring provide the same standard of care as that offered in traditional in‐clinic visits?	‐ Yes 83%
	‐ No 17%

### 3.2 Activation and in‐hospice management of RM

The median number of activations per center was 15 [25th‐75th percentile: 9–33]. The median relative increase was 60% [25th‐75th percentile: 15–100]. The relative increase of activations was 12% [25th‐75th percentile: 10–55] in centers that remotely monitored 50 or more patients via the LATITUDE system before the lockdown period, and 100% [25th‐75th percentile: 38–113] in centers that remotely monitored less than 50 patients (*p* = .004). By the end of the lockdown period, 50 or more patients were being remotely monitored via the LATITUDE system in 25 centers, and less than 50 in 24 centers. Twenty‐eight (2%) patients refused the system. In addition, 376 patients underwent de‐novo Boston Scientific CIED implantation or generator exchange during the lockdown period. They received the LATITUDE communicator before hospital discharge and were not included in the present analysis.

**FIGURE 1:**
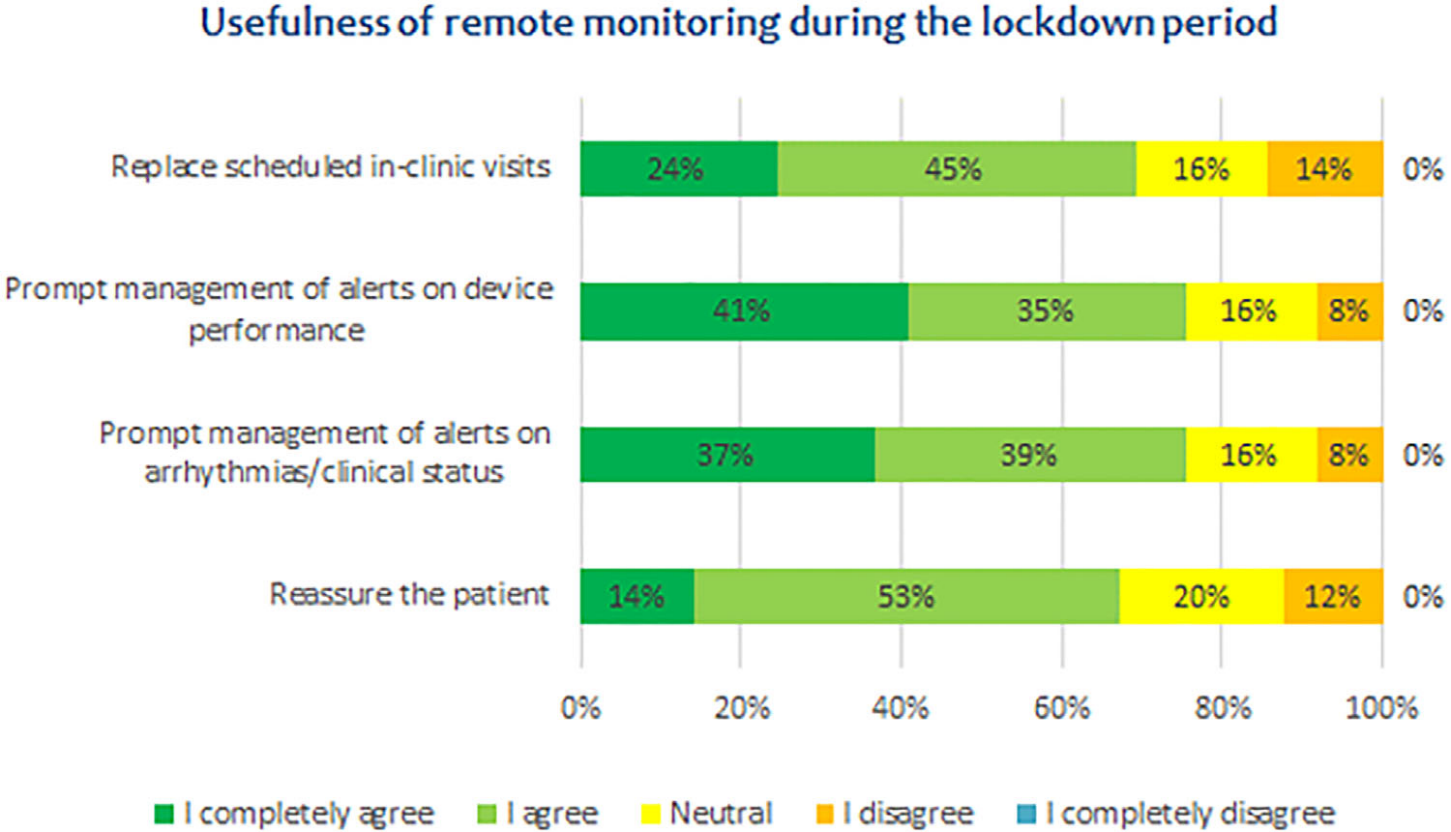
Clinicians' opinions of the usefulness of remote monitoring (RM) during the lockdown period [Color figure can be viewed at wileyonlinelibrary.com]

The management of RM during the COVID‐19 pandemic did not change significantly. In most centers, the frequency of scheduled remote interrogations remained unchanged and no changes in alert programming were made. By contrast, in many centers, in‐clinic evaluations were canceled or performed only in specific situations or for patients at higher risk. In order to coordinate the home delivery of communicators, remote training and activations, 20% of centers required additional staff. The vast majority of respondents agreed that RM was effective in managing patients during the lockdown period, and that it allowed them to provide the same care as that offered by traditional in‐clinic visits (Table 1). Figure 1 reports the answers of the physicians regarding the usefulness of RM during the lockdown period. Most physicians stated that RM allowed them to replace scheduled in‐clinic visits for device follow‐up and to promptly manage alerts concerning device performance, arrhythmias and clinical status/heart failure. They also generally agreed that offering the RM service gave their patients a sense of reassurance.

### 3.3 Home delivery of the communicator

**FIGURE 2:**
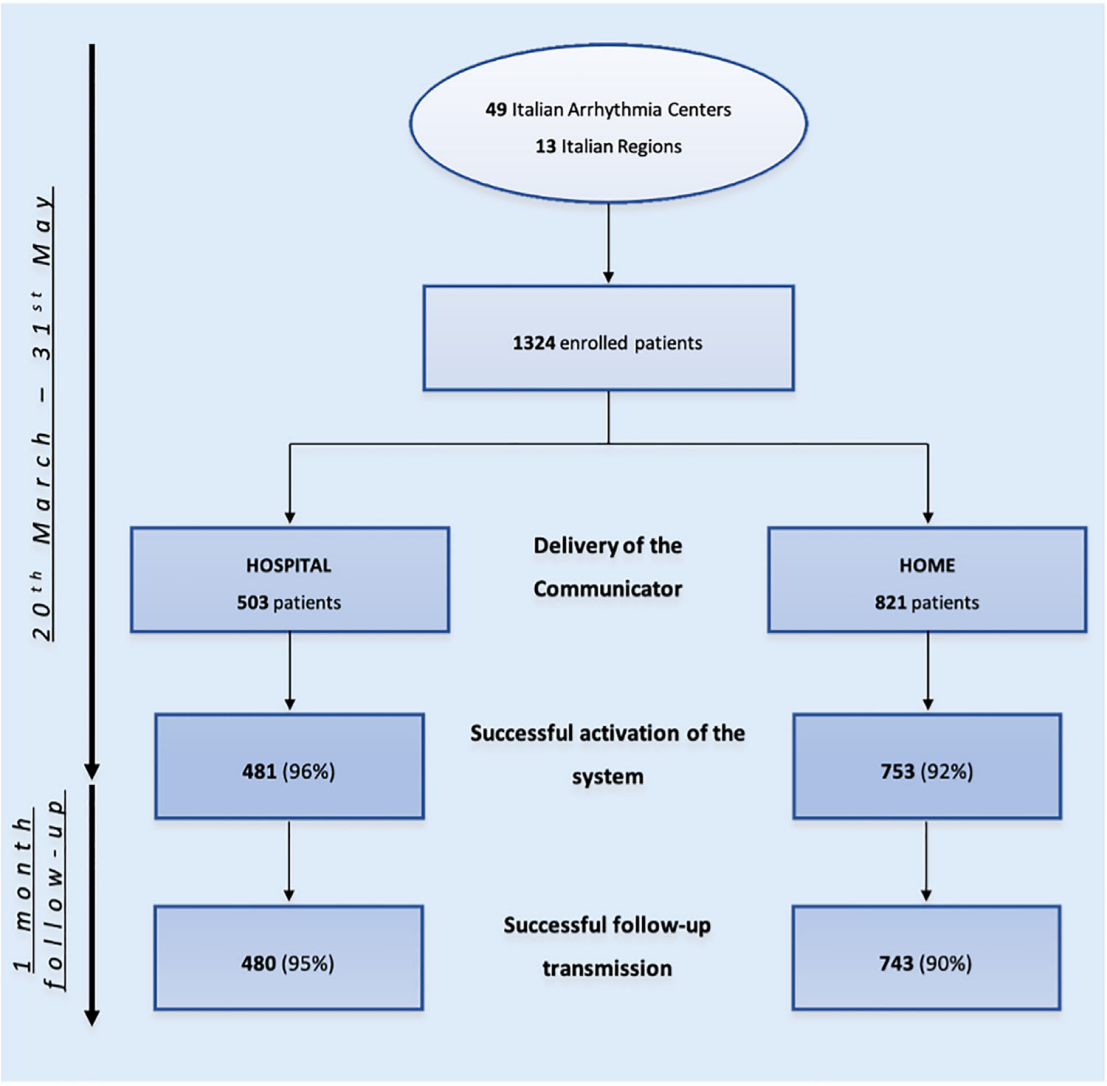
Delivery of the communicator and activation rate between two groups (home vs. office) [Color figure can be viewed at wileyonlinelibrary.com]

As depicted in Figure 2, of the 1324 patients who agreed to receive the RM system, 503 (38%) went to the hospital to receive the system and the necessary instructions, while the remaining 821 (62%) received the communicator at home, and it was activated remotely. Patients were contacted by a physician in 67% of cases and by a nurse in the remaining 33%, in order to be trained in the activation process and the routine use of the communicator. After the first contact with the patient, activation required one additional call in 49% of cases and more than one in 39%; in 98% of cases, the phone contacts took place after the delivery of the communicator. To carry out the activation process, 12% of patients contacted the Boston Scientific dedicated technical support call center.

**FIGURE 3:**
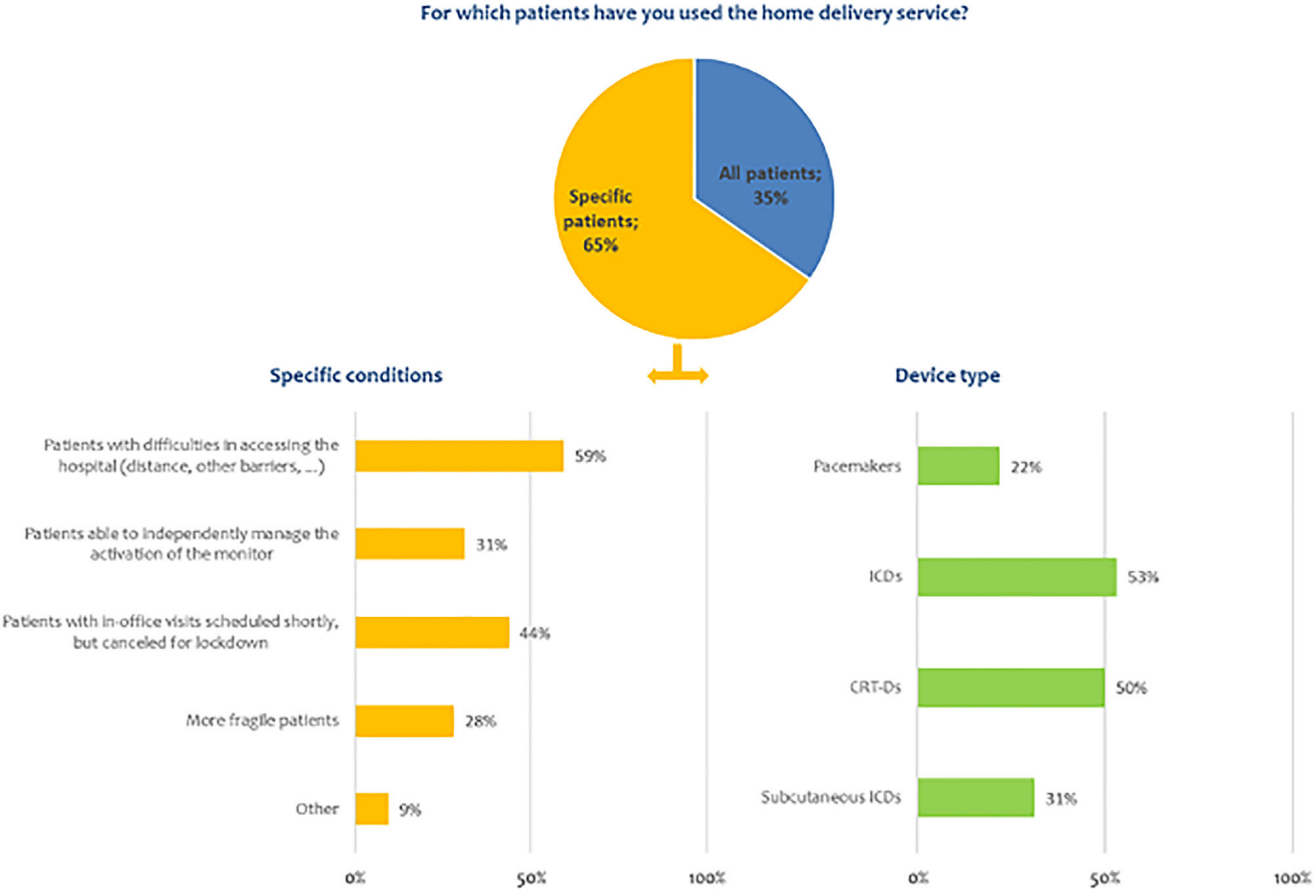
Use of the home‐delivery service at the centers that responded to the survey [Color figure can be viewed at wileyonlinelibrary.com]

Overall, the median time needed to complete the activation process was 15 min [25th‐75th percentile: 10–20]. The median number of communicators delivered at home was 11 per center [25th‐75th percentile: 3–20] and home‐delivered communicators accounted for 80% of all new activations of RM [25th‐75th percentile: 50–100]. The proportion of home‐delivered communicators was 50% [25th‐75th percentile: 29–83] in centers situated in regions with a high incidence of COVID‐19 cases, 72% [25th‐75th percentile: 51–96] in regions with an intermediate incidence, and 89% [25th‐75th percentile: 71–100] in regions with a low incidence (*p* = .175). The proportion of home‐delivered communicators was 59% [25th‐75th percentile: 29–78] in centers that remotely monitored 50 or more patients via the LATITUDE system before the lockdown period, and 91% [25th‐75th percentile: 55–100] in centers that remotely monitored less than 50 patients (*p* = .005). In the case of home delivery of communicators, the signature of the patient's informed consent document was obtained by e‐mail in 63% of cases and at the hospital in 16% of cases; consent was obtained by telephone in 16% of cases and by other methods in the remaining 5% of cases. Figure 3 shows the use of the home‐delivery service, broken down by type of patient and device, at the centers that responded to the survey. The majority of centers preferred to assign a home‐delivered communicator to specific subgroups of patients, that is., patients with more complex devices–implantable defibrillators (ICD) or cardiac resynchronization therapy (CRT) ‐, patients with difficulties in accessing the hospital or patients with visits scheduled shortly.

### 3.4 Activation rate and follow‐up transmission

Of the 821 patients who received the communicator at home and who were remotely trained to use the system, 753 (92%) were able to perform a manual transmission in order to complete the correct activation of the system (Figure 2). The median rate of correct activation was 100% [25th‐75th percentile: 100–100] in centers that remotely monitored 50 or more patients via the LATITUDE system before the lockdown period, and 95% [25th‐75th percentile: 86–100] in centers that remotely monitored less than 50 patients (*p* = .050). At the time when the questionnaire was completed, 743 (90%) communicators were regularly transmitting data. Interrupted transmissions were due to unplugged communicator or because the patient was not compliant with prescribed use, not allowing to establish telemetry sessions. Of the 503 communicators delivered during in‐clinic visits, 481 (96%, *p* = .006) were successfully activated and 480 (95%, *p* = .001) continued to transmit regularly. Table 2 reports clinicians' feedback on the home‐delivery service. The service was generally deemed useful in facilitating the activation of RM during the COVID‐19 pandemic and possibly beyond.

**TABLE 2:** Clinician feedback on the home‐delivery service

Clinician feedback on the home‐delivery service	
Was the home‐delivery service useful in managing patients during the lockdown period?	‐ Yes 96%
	‐ No 4%
Is home delivery an efficient method for activating remote monitoring?	‐ Yes 94%
	‐ No 6%
In your opinion, did the patients appreciate this mode of delivery?	‐ Yes 98%
	‐ No 2%
If available, would you continue to use home delivery in the future?	‐ Yes 92%
	‐ No 8%
Do you think there is a need for more structured support by Boston Scientific?	‐ Yes^a^ 59%
	‐ No 41%

^a^ Information material and digital tools (videos, dedicated apps, etc.) to support patients in installing and verifying system operation (67% of the answers).

## 4 DISCUSSION

This multicenter study examined the utility and feasibility of home delivery of communicators for RM. Its main findings are:

‐ Reorganization of RM enrollment during the lockdown period was easily achieved in a large population of CIED patients; services of comparable quality were provided, and no additional personnel was required in 78% of the centers.
‐ Although a higher activation rate was observed when the communicator was delivered during an in‐clinic visit (95% vs. 92%, *p* = .001), the majority (92%) of patients who received the communicator at home were able to correctly finalize activation of the system. Moreover, thanks to the trans‐telephonic technical support, 90% of communicators were regularly transmitting data at the time of questionnaire completion.
‐ The home‐delivery service was especially adopted for patients with ICD or CRT, those with difficulties in accessing the hospital (59%) and those whose scheduled office visit was canceled because of the lockdown (44%).
‐ Clinician feedback on the home‐delivery service was very satisfying: the service was deemed to be an efficient and useful means of managing patients during the lockdown period. In addition, 92% of clinicians were interested in continuing the use of this service.

The spread of the COVID‐19 epidemic required a rapid response with regard to in‐hospital activity.^1^, ^2^ The primary modes of disease prevention recommended by the Center for Disease Control have involved limiting exposure and social distancing. To comply with these measures, the healthcare system has had to postpone elective procedures and non‐essential follow‐up visits, implement virtual interactions and adopt new work procedures in order to provide excellent care.^10^, ^11^, ^12^, ^13^, ^14^ This rapid reorganization has also affected CIED patients, for whom RM has proved able to replace in‐person visits.^15^, ^16^, ^17^, ^18^, ^19^, ^20^ As reported in the Heart Rhythm Society COVID‐19 Task Force and ESC COVID‐19 guidelines, in order to maintain a high degree of safety and limit face‐to‐face interactions, an extensive use of telehealth technologies has been necessary.^11^

The safety and efficacy of RM was established a decade ago by the landmark TRUST trial, in which RM with automatic daily surveillance of ICD was able to detect actionable events more rapidly than conventional in‐office visits.^15^ As also reported in several studies, not only does RM play a central role in preventing hospitalizations and improving survival and quality of life in patients with CIEDs, it is also a cost‐effective alternative to in‐person evaluations.^16^, ^17^, ^18^, ^19^, ^20^, ^21^, ^22^, ^23^, ^24^, ^25^ Overall, in the context of the COVID‐19 emergency, the improvement of RM coverage has facilitated continuous patient assistance and significantly reduced the risk of virus transmission among both more vulnerable populations and healthcare providers.^26^, ^27^, ^28^

Our Italian multicenter study involved patients from different centers located in areas with different degrees of exposure to COVID‐19, with 29 (59%) of 49 centers being located in regions with a moderate‐high incidence of COVID‐19 cases. A large number of patients were rapidly introduced to RM: the LATITUDE system was activated in 1324 patients, 821 (62%) of whom received the communicator at home, while 503 (38%) received it in hospital. Prior to the lockdown, most centers (91%) monitored fewer than 50 patients via the LATITUDE System, while by the end of the enrollment period 25 centers (50%) were monitoring at least 50 patients via this system. The increased number of patients enrolled was achieved thanks to the competence of the staff and their ability to reinforce patients’ feeling of being constantly assisted, both technically and clinically. Despite the higher number of patients remotely monitored, the centers did not make substantial changes to the use and organization of RM; consequently, no significant increase in resources was necessary. Furthermore, the median time required to complete the activation of RM was 15 min per patient; this was comparable to the time usually needed for in‐office activation, as reported in the HomeGuide Registry.^29^ Moreover, only 22% of the centers required additional staff, indicating that RM is not only effective in preventing hospitalizations but also a cost‐effective alternative to in‐hospital follow‐up.^30^, ^31^, ^32^, ^33^ Finally, in this emergency setting, informed consent to home delivery of communicators was obtained from patients by means of e‐mail or telephone, in order to avoid personal contacts. These strategies could be maintained in the future through adequate and safe online data storage or sharing systems, thereby facilitating the traceability and management of consent documents.

The results of our survey reveal general approval of RM and of the usefulness of the new service of home delivery; indeed, very few patients refused the system (n = 28, 2%). As expected, centers preferred to assign a home‐delivered communicator to specific subgroups of patients (i e., ICD, CRT‐D, patients with difficulties in accessing the hospital or those with visits scheduled shortly, in order to protect these subpopulations at higher risk of COVID‐19‐related complications and mortality. The activation of RM required additional trans‐telephonic support and, in 12% of cases, contact with the Boston Scientific dedicated technical support center. Nevertheless, patient acceptance was high, and 92% of patients who received the communicator at home were able to perform a manual transmission. Moreover, 743 (90%) communicators were regularly transmitting data at the time of questionnaire completion. There may be several explanations for why the rate of RM activation was lower in the home‐delivery group than the rate recorded in a single‐center experience or when the communicator was delivered in hospital: the heterogeneity of the various centers included, the short period of observation, and individual patients' compliance or preference.^13^ The observation that the percentage of correct activations was higher in centers with previous greater RM activity, suggests that the experience of the center may play a role in patient training. These considerations show that the procedure needs to be better planned in order to be applied in all centers in a non‐emergency setting. This is relevant, as patient training has an important role in the perception and acceptance of RM^34^ and in the continuity of monitoring, which is also known to be linked to patient outcome.^35^

As emerged from the questionnaire, the home‐delivery service received positive feedback from clinicians, 96% of whom considered it useful in order to manage patients during the COVID‐19 pandemic, while 92% stated that they like to continue it beyond the lockdown. Only 4% of clinicians claimed that RM was not a useful means of patient management and 6% thought that home delivery was not efficient for RM activation. These negative views could probably be improved through more consolidated organization or by providing better assistance by medical and technical staff.

### 4.1 Limitations

Our findings are affected by potential limitations. The project was limited to a single RM platform, thus our results may not be applicable to other systems. Moreover, the participation in the initiative and in the present survey was voluntary, and this may have introduced biases. In addition, we cannot exclude possible differences in the implementation of the initiative among centers, with an impact on the degree of success. However, the overall success of the initiative is reassuring.

## 5 CONCLUSION

The increased use of a RM protocol will reduce risks for both providers and patients, while maintaining high‐quality care. Home delivery of the communicator proved to be a successful approach to system activation, which is a major determinant of effective RM, and both clinicians and patients agreed on the usefulness of this model. Similarly, remotely training the patient to use the system seemed feasible.

## AUTHOR CONTRIBUTIONS

Conception and design: Magnocavallo M, Bernardini A, Mariani MV, Lavalle C, Valsecchi S, Campari M; data collection: Marini M, Nicosia A, Adduci C, Rapacciuolo A, Saporito D, Grossi S, Santarpia G, Vaccaro P, Rordorf R, Pentimalli F; analysis and interpretation of data: Magnocavallo M, Valsecchi S, Mariani MV; drafting: Magnocavallo M, Bernardini A, Valsecchi S; critical review for important intellectual content: Valsecchi S, Campari M, Giunta G, Lavalle C.

## CONFLICTS OF INTEREST

Monica Campari and Sergio Valsecchi are employees of Boston Scientific. The other authors report no conflicts.

## Supporting information

Supplementary information

## Data Availability

The data that support the findings of this study are not openly available due to reasons of sensitivity and are available from the corresponding author upon reasonable request.

## References

[1] Wang D , Hu B , Hu C , et al. Clinical characteristics of 138 hospitalized patients with 2019 novel coronavirus‐infected pneumonia in Wuhan, China. JAMA. 2020;323:1061. 32031570 10.1001/jama.2020.1585 PMC7042881

[2] Zhou P , Yang X‐L , Wang X‐G , et al. A pneumonia outbreak associated with a new coronavirus of probable bat origin. Nature. 2020;579:270‐273. 32015507 10.1038/s41586-020-2012-7 PMC7095418

[3] Zhu N , Zhang D , Wang W , et al. A novel coronavirus from patients with pneumonia in China. N Engl J Med. 2019;382:727‐733. 10.1056/NEJMoa2001017 PMC7092803 31978945

[4] Guan W , Ni Z , Hu Y , et al. Clinical characteristics of coronavirus disease 2019 in China. N Engl J Med. 2020;382:1708‐1720. 32109013 10.1056/NEJMoa2002032 PMC7092819

[5] Della Rocca DG , Magnocavallo M , Lavalle C , et al. Evidence of systemic endothelial injury and microthrombosis in hospitalized COVID‐19 patients at different stages of the disease. J Thromb Thrombolysis. 2020;51:571‐576. 33156441 10.1007/s11239-020-02330-1 PMC7645404

[6] Bedford J , Enria D , Giesecke J , et al. COVID‐19: towards controlling of a pandemic. Lancet. 2020;395:1015‐1018. 32197103 10.1016/S0140-6736(20)30673-5 PMC7270596

[7] Fulchand S . Covid‐19 and cardiovascular disease. BMJ. 2020;369:m1997. 32434891 10.1136/bmj.m1997

[8] Boriani G , Palmisano P , et al, AIAC Ricerca Network Investigators˘ . Impact of COVID‐19 pandemic on the clinical activities related to arrhythmias and electrophysiology in Italy: results of a survey promoted by AIAC (Italian Association of Arrhythmology and Cardiac Pacing). Intern Emerg Med. 2020;15:1445‐1456. 32889687 10.1007/s11739-020-02487-w PMC7474489

[9] Severino P , D'Amato A , Saglietto A , et al. Reduction in heart failure hospitalization rate during coronavirus disease 19 pandemic outbreak. ESC Heart Fail. 2020;7:4182‐4188. 10.1002/ehf2.13043 PMC7754919 33094929

[10] Mohanty S , Lakkireddy D , Trivedi C , et al. Creating a safe workplace by universal testing of SARS‐CoV‐2 infection in asymptomatic patients and healthcare workers in the electrophysiology units: a multi‐center experience. J Interv Card Electrophysiol. 2020;1:1‐6. 10.1007/s10840-020-00886-9 PMC7529320 33006086

[11] Lakkireddy DR , Chung MK , Gopinathannair R , et al. Guidance for cardiac electrophysiology during the COVID‐19 pandemic from the Heart Rhythm Society COVID‐19 task force; electrophysiology section of the american college of cardiology; and the electrocardiography and arrhythmias committee of the council on clinical cardiology. Heart Rhythm Am Heart Assoc. 2020;17:e233‐e241. 10.1016/j.hrthm.2020.03.028 PMC7118697 32247013

[12] Varma N , Marrouche NF , Aguinaga L , et al. HRS/EHRA/APHRS/LAHRS/ACC/AHA worldwide practice update for telehealth and arrhythmia monitoring during and after a pandemic. J Am Coll Cardiol. 2020;76:1363‐1374. 32534936 10.1016/j.jacc.2020.06.019 PMC7289088

[13] Piro A , Magnocavallo M , Della Rocca DG , et al. Management of cardiac implantable electronic device follow‐up in COVID‐19 pandemic: lessons learned during Italian lockdown. J Cardiovasc Electrophysiol. 2020;31:2814‐2823.jce.14755. 32954600 10.1111/jce.14755 PMC7646650

[14] Hollander JE , Carr BG . Virtually perfect? Telemedicine for Covid‐19. N Engl J Med. 2020;382:1679‐1681. 32160451 10.1056/NEJMp2003539

[15] Varma N , Epstein AE , Irimpen A , Schweikert R , Love C . Efficacy and safety of automatic remote monitoring for implantable cardioverter‐defibrillator follow‐up: the Lumos‐T safely reduces routine office device follow‐up (TRUST) trial. Circulation. 2010;122:325‐332. 20625110 10.1161/CIRCULATIONAHA.110.937409

[16] Slotwiner D , Varma N , Akar JG , et al. HRS expert consensus statement on remote interrogation and monitoring for cardiovascular implantable electronic devices. Heart Rhythm. 2015;12:e69‐e100. 25981148 10.1016/j.hrthm.2015.05.008

[17] Heidbuchel H , Hindricks G , Broadhurst P , et al. EuroEco (European Health Economic Trial on home monitoring in ICD patients): a provider perspective in five European countries on costs and net financial impact of follow‐up with or without remote monitoring. Eur Heart J. 2015;36:158‐169. 25179766 10.1093/eurheartj/ehu339 PMC4297469

[18] Ricci RP , Morichelli L , Quarta L , et al. Long‐term patient acceptance of and satisfaction with implanted device remote monitoring. Europace. 2010;12:674‐679. 20200019 10.1093/europace/euq046

[19] Akar JG , Bao H , Jones P , et al. Use of remote monitoring of newly implanted cardioverter‐defibrillators: insights from the patient related determinants of ICD remote monitoring (PREDICT RM) study. Circulation. 2013;128:2372‐2383. 24043302 10.1161/CIRCULATIONAHA.113.002481

[20] Zanotto G , D'Onofrio A , Della Bella P , et al. Organizational model and reactions to alerts in remote monitoring of cardiac implantable electronic devices: a survey from the Home Monitoring Expert Alliance project. Clin Cardiol. 2019;42:76‐83. 30421438 10.1002/clc.23108 PMC6436519

[21] Della Rocca DG , Albanese M , Placidi F , et al. Feasibility of automated detection of sleep apnea using implantable pacemakers and defibrillators: a comparison with simultaneous polysomnography recording. J Interv Card Electrophysiol. 2019;56:327‐333. 31646429 10.1007/s10840-019-00631-x

[22] Crossley GH . Further evidence that remote monitoring is cost‐effective: it's time for all to adopt. Heart Rhythm. 2017;14:58. 27670630 10.1016/j.hrthm.2016.09.023

[23] Luzi M , De Simone A , Leoni L , et al. Remote monitoring for implantable defibrillators: a Nationwide survey in Italy. Interact J Med Res. 2013;2:e27. 24055720 10.2196/ijmr.2824 PMC3786126

[24] Maines M , Catanzariti D , Angheben C , Valsecchi S , Comisso J , Vergara G . Intrathoracic impedance and ultrasound lung comets in heart failure deterioration monitoring: intrathoracic impedance and ultrasound lung comets. Pacing Clin Electrophysiol. 2011;34:968‐974. 21477028 10.1111/j.1540-8159.2011.03072.x

[25] Forleo GB , Tesauro M , Panattoni G , et al. Impact of continuous intracardiac ST‐segment monitoring on mid‐term outcomes of ICD‐implanted patients with coronary artery disease. Early results of a prospective comparison with conventional ICD outcomes. Heart. 2012;98:402‐407. 22115985 10.1136/heartjnl-2011-300801

[26] De Larochellière H , Champagne J , Sarrazin J , et al. Findings of remote monitoring of implantable cardioverter defibrillators during the COVID‐19 pandemic. Pacing Clin Electrophysiol. 2020;43:1366‐1372. 33021739 10.1111/pace.14086 PMC7675613

[27] Iacopino S , Placentino F , Colella J , et al. Remote monitoring of cardiac implantable devices during COVID‐19 outbreak: “keep people safe” and “focus only on health care needs. Acta Cardiologica. 2020;18:1‐4. 10.1080/00015385.2020.1847459 33203312

[28] Auricchio A , Conte G , Demarchi A , et al. Challenges in activation of remote monitoring in patients with cardiac rhythm devices during the coronavirus (COVID‐19) pandemic. Int J Cardiol. 2021;328:247‐249. 33278416 10.1016/j.ijcard.2020.11.063 PMC7709476

[29] Ricci RP , Morichelli L , D'Onofrio A , et al. Manpower and outpatient clinic workload for remote monitoring of patients with cardiac implantable electronic devices: data from the homeguide registry: manpower of cardiac device home monitoring. J Cardiovasc Electrophysiol. 2014;25:1216‐1223. 24964380 10.1111/jce.12482

[30] Sequeira S , Jarvis CI , Benchouche A , Seymour J , Tadmouri A . Cost‐effectiveness of remote monitoring of implantable cardioverter‐defibrillators in France: a meta‐analysis and an integrated economic model derived from randomized controlled trials. EP Europace. 2020;22:1071‐1082. 10.1093/europace/euaa082 32424395

[31] Hummel JP , Leipold RJ , Amorosi SL , et al. Outcomes and costs of remote patient monitoring among patients with implanted cardiac defibrillators: an economic model based on the PREDICT RM database. J Cardiovasc Electrophysiol. 2019;30:1066‐1077. 30938894 10.1111/jce.13934 PMC6850124

[32] Burri H , Sticherling C , Wright D , Makino K , Smala A , Tilden D . Cost‐consequence analysis of daily continuous remote monitoring of implantable cardiac defibrillator and resynchronization devices in the UK. Europace. 2013;15:1601‐1608. 23599169 10.1093/europace/eut070 PMC3810620

[33] Della Rocca DG , Santini L , Forleo GB , et al. Novel perspectives on arrhythmia‐induced cardiomyopathy: pathophysiology, clinical manifestations and an update on invasive management strategies. Cardiol Rev. 2015;23:135‐141. 25133468 10.1097/CRD.0000000000000040

[34] Laurent G , Amara W , Mansourati J , et al. Role of patient education in the perception and acceptance of home monitoring after recent implantation of cardioverter defibrillators: the EDUCAT study. Arch Cardiovas Dis. 2014;107:508‐518. 10.1016/j.acvd.2014.06.009 25218008

[35] Akar JG , Bao H , Jones PW , et al. Use of remote monitoring is associated with lower risk of adverse outcomes among patients with implanted cardiac defibrillators. Circ Arrhythm Electrophysiol. 2015;8:1173‐1180. 26092577 10.1161/CIRCEP.114.003030

