# Supplementary material for: Home delivery of the communicator for remote monitoring of cardiac implantable devices: A multicenter experience during the covid‐19 lockdown
Source: Pacing Clin Electrophysiol. 2021 May 15;44(6):995–1003. doi: 10.1111/pace.14251 (PMC8207054; doi:10.1111/pace.14251)
Supplement: Supplementary file 1 — Supplementary information [file PACE-44-995-s001.docx]

**Supplementary material**

**LATITUDE at home Campaign**

1. N. Devices (PM, ICD, CRT-D, S-ICD) remotely monitored before the lockdown period

2. N. Device (PM, ICD, CRT-D, S-ICD) remotely monitored to date

3. N. of Latitude communicators delivered to patients’ homes during lockdown period:

- N. of successful activations
- N. of systems regularly transmitting to date

4. N. of Latitude communicators delivered in hospital during lockdown period:

- N. of successful activations
- N. of systems regularly transmitting to date

5. For which patients have you used the home-delivery service of the Latitude Communicator?

- All patients
- Specific patients

6. If you answered “Specific patients”, please specify the type(s) of patients

- - PM
  - ICD
  - CRT-D
  - S-ICD
  - Patients with difficulties in accessing the hospital (distance, barriers,…)
  - Patients able to independently manage the activation of the monitor
  - Patients with in-office visits scheduled shortly, but canceled for lockdown
  - More fragile patients
  - Other, specify

7. How many patients refused home delivery of the remote monitoring communicator?

8. For what reason did patients refuse home delivery?

9. Who supported patients during the home-delivery process and Latitude communicator activation?

- Physician
- Nurse/technician
- Other, specify

10. To perform this new delivery service, did you need additional staff?

- Yes
- No

If yes, please specify

11. How long, on average, did it take to manage each single activation (presentation, consent, explanation of remote monitoring and activation)

12. Since the first contact to present the system and obtain consent for home delivery of the system, how many additional contacts have been required for each patient (explanation, clarification, technical problems,…)

- None
- 1
- 2
- >2

13. When were these additional contacts made?

- Before the delivery of the Latitude communicator
- After the delivery of the Latitude communicator

14. How many patients have contacted you for assistance?

15. How many patients have contacted the Boston Scientific Assistance service?

16. How did you manage the informed consent of patients?

- Via mail/telematic system
- Phone contact recording
- Other, specify

17. Was the home-delivery service useful in managing patients during the lockdown period?

- Yes
- No

18. Is home delivery an efficient method for activating remote monitoring?

- Yes
- No

19. In your opinion, did the patients appreciate this mode of delivery?

- Yes
- No

20. If available, would you continue to use home delivery in the future?

- Yes
- No

21. Do you think there are any aspects that could be improved in the process of sending the communicator directly to the patient?

- Yes
- No

If yes, please specify

22. Do you think there is a need for more structured support by Boston Scientific?

- No, the service is enough
- Yes, information material and digital tools (videos, dedicated apps, etc.) to support patients in installing and verifying system operation
- Yes, specify

**Remote Monitoring**

Before the lockdown due to the Covid-19 pandemic:

23. Who was involved in the management of remote monitoring (follow-up or alert management):

- Only physician
- Nurse/technician and physician in the case of necessity (triage)

24.What alerts did you usually program?

- Device performance
- Clinical status
- Yellow alerts
- Red alerts

During the lockdown period:

25.Was the frequency of scheduled remote interrogations modified?

- Yes, increased
- Yes, decreased
- No

Specify

26.Was the programming of alerts modified?

- Yes
- No

Specify new alerts programmed

27. In-clinic scheduled visits were:

- All canceled
- All confirmed
- Performed only in specific situations

Specify

28. How many unscheduled remote transmissions (due to alerts) have been managed?

29. How many patients needed unscheduled in-clinic visits (related to the device, arrhythmias or heart failure)?

30. Did the remote monitoring system require additional staff?

- Yes
- No

If yes, please specify

31. Do you think that remote monitoring was effective in managing patients during the lockdown period?

- Yes
- No

32. Do you think that remote monitoring allowed you to provide the same standard of care as that offered in traditional in-clinic visits?

- Yes
- No

33. Following your experience, how do you evaluate the usefulness of the remote monitoring system during the lockdown period? (Not useful, Quite useful, Useful, Very useful, Necessary)

- To replace scheduled visits
- To reassure the patient
- To promptly manage alerts due to device malfunctions
- To promptly manage alerts due to arrhythmias and heart failure

**APPENDIX: List of participating centers**

A.O.U. Federico II (Naples) - Dr. Antonio Rapacciuolo

A.O.U. Mater Domini (Catanzaro) - Dr. Giuseppe Santarpia

Azienda Ospedaliera Cannizzaro (Catania) - Dr. Marco Lisi

Campus Biomedico (Rome) - Dr. Danilo Ricciardi

Casa Sollievo della Sofferenza (San Giovanni Rotondo) - Dr. Raimondo Massaro

Grande Ospedale Metropolitano (Reggio Calabria) - Dr. Antonio Pangallo

IRCCS Centro Neurolesi (Messina) - Dr. Giuseppe Picciolo

IRCCS Policlinico S. Matteo (Pavia) - Dr. Roberto Rordorf

IRCCS S. Cuore Don Calabria (Negrar) - Dr. Giulio Molon

Istituto Clinico San Rocco (Ome) - Dr. Armando Hardini

Ospedale San Paolo (Savona) - Dr. Luca Bacino

Ospedale della Misericordia (Grosseto) - Dr. Gennaro Miracapillo

Ospedale di Rivoli (Rivoli) - Dr. Antonio Mazza

Ospedale F. Spaziani (Frosinone) - Dr.ssa Giovanna Giubilato

Ospedale Giovanni Paolo II (Ragusa) - Dr. Antonino Nicosia

Ospedale Grassi (Ostia) - Dr. Karim Mahfouz

Ospedale Infermi (Rimini) - Dr. Davide Saporito

Ospedale Jazzolino (Vibo Valentia) - Dr. Giuseppe Carullo

Ospedale Macchi (Varese) - Dr. Fabrizio Caravati

Ospedale Maggiore (Modica) - Dr.ssa Sabina Ficili

Ospedale Mauriziano (Turin) - Dr. Stefano Grossi

Ospedale Nostra Signora di Bonaria (San Gavino Monreale) - Dr. Roberto Floris

Ospedale Pertini (Rome) - Dr.ssa Veronica Rizzo

Ospedale Pineta Grande (Naples) - Dr. Luigi Argenziano

Ospedale S. Giuseppe da Copertino (Copertino) - Dr.ssa Elena Marino

Ospedale S. M. Annunziata (Bagno a Ripoli) - Dr. Simone Vignini

Ospedale S. Maria del Carmine (Rovereto) - Dr. Giancarlo Tomasi

Ospedale San Luca (Lucca) - Dr. Davide Giorgi

Ospedale San Martino (Genoa) - Dr. Paolo Sartori

Ospedale Sant’Andrea (Rome) - Dr.ssa Carmen Adduci

Ospedale Santa Chiara (Trento) - Dr.ssa Silvia Quintarelli

Ospedale Santa Maria Nuova (Florence) - Dr. Alessandro Paoletti Perini

Ospedale Sant'Andrea (La Spezia) - Dr.ssa Sandra Badolati

Ospedale Sant'Anna e San Sebastiano (Caserta) - Dr. Agostino Mattera Iacono

Ospedale Santo Spirito (Rome) - Dr. Andrea Porzio

Ospedale Versilia (Lido di Camaiore) - Dr. Gianluca Solarino

Ospedale Villa Scassi (Genoa) - Dr. Mattia Laffi

Ospedale Vito Fazzi (Lecce) - Dr. Ennio Carmine Pisanò

Ospedale Vittorio Emanuele (Catania) - Dr. Antonino Tosto

Ospedale Vittorio Emanuele (Gela) - Dr. Carmelo Tonelli

P.O Muscatello (Augusta) - Dr. Giuseppe Busacca

P.O. Cervello (Palermo) - Dr.ssa Paola Vaccaro

P.O. S Maria Delle Grazie (Pozzuoli) - Dr. Pasquale Nocerino

P.O. Pellegrini (Naples) - Dr.ssa Ilaria De Crescenzo

Policlinico Casilino (Rome) - Dr. Ermenegildo De Ruvo

Policlinico Umberto I (Rome) - Dr. Carlo Lavalle

UOD Elettrofisiologia - Osp Monaldi (Naples) - Dr. Valter Bianchi

Villa dei Fiori (Acerra) - Dr. Paolo Gallo

Villa Sofia (Palermo) - Dr. Antonino Mignano
